# Sumatriptan-Induced Coronary Artery Vasospasm Leading to Acute ST Elevation Myocardial Infarction

**DOI:** 10.7759/cureus.80920

**Published:** 2025-03-20

**Authors:** Ramesh Acharya, Sachin Sapkota, Lela Ruck, Mehran Abolbashari

**Affiliations:** 1 Internal Medicine, The Hospitals of Providence-Transmountain, Paul L. Foster School of Medicine, Texas Tech University Health Sciences Center El Paso, El Paso, USA; 2 Cardiovascular Medicine, Center of the Heart, Providence Medical Partners, El Paso, USA

**Keywords:** acute coronary syndrome, anomalous right coronary artery, case report, drug reaction, electrocardiogram (ecg/ekg), st-elevation myocardial infarction (stemi), sumatriptan

## Abstract

Sumatriptan, a 5-HT1 receptor agonist, is a highly effective treatment for migraines, replacing ergotamine as the mainstay therapy. However, its use has been associated with coronary vasospasm due to the presence of 5-HT1-like receptors in coronary arteries, which can lead to acute myocardial infarction (AMI). This report presents the case of a 50-year-old female patient with a history of hypertension treated on lisinopril with no previous cardiac evaluations and no prior history of chest pain, a family predisposition to coronary artery disease who presented with chest pain and experienced ischemic electrocardiographic changes following a sumatriptan injection. Despite a normal baseline electrocardiogram and non-obstructive coronaries on coronary angiography, the patient reported severe chest pain after sumatriptan administration, which resolved with nitrate treatment. This case highlights the possibility of AMI following sumatriptan use with non-obstructive coronaries and the need for careful patient evaluation for coronary risk factors prior to prescribing sumatriptan. The patient did not report any episodes of chest pain on the initial clinic evaluation post discharge from the hospital. Patients should be informed of the risk of myocardial infarction and advised to seek medical attention if they experience chest pain. It underscores the importance of thorough risk assessment and patient education when considering sumatriptan for migraine treatment.

## Introduction

For many years, ergotamine was the mainstay treatment for migraines; however, sumatriptan, a 5-HT1 receptor agonist, now provides a novel and highly effective treatment for migraines [1]. Sumatriptan has a relatively short half-life of about two hours, with most of the drug excreted as inactive metabolites in the urine and common side effects including paraesthesia, flushing, dizziness, and chest discomfort. This vasospasm occurs because sumatriptan, a selective agonist of 5-HT1B and 5-HT1D receptors, can cause vasoconstriction in cranial blood vessels and coronary arteries. 

Primarily, 5HT1 receptors inhibit adenylate cyclase and regulate mood and vascular tone. In contrast, 5-HT2 receptors activate phospholipase C and are involved in smooth muscle contraction and modulation of perception and mood. Acute myocardial infarction (AMI) occurs due to compromised blood flow and oxygen to heart muscles. Sumatriptan should not affect most vascular beds in the body because they exhibit 5-HT1B-mediated vasoconstriction. However, recent studies have identified 5-HT1-like receptors in coronary arteries [2-5]. While 5-HT2 receptors mainly mediate contractions induced by 5-HT, the 5-HT1-like receptor also contributes to coronary vasospasm [3-5]. 

Macintyre et al. studied 10 patients undergoing diagnostic coronary arteriography and found that subcutaneous sumatriptan injection significantly reduced mean absolute coronary artery diameter, and vasoconstrictor effects were seen in the pulmonary, systemic, and coronary circulations, which were not associated with clinically significant ischemia [5]. One study examined human-isolated coronary arteries from the explanted hearts of four male patients undergoing heart transplantation, revealing that 5-HT1-like receptors play a role in the contractile response to 5-HT [6]. There is considerable evidence of EKG changes indicative of transient coronary ischemia after the use of sumatriptan [7,8]. O’Quinn et al., in an open-label prospective trial, studied 12,339 typical migraineurs for up to 12 months each, where 36 patients reported symptoms or signs consistent with dysrhythmias, heart failure, angina, myocardial infarction (MI), or cardiomyopathy, with three cases of MIs and six instances of angina; however, none of the three reported MIs related to the triptan use [9].

While sufficient evidence demonstrates sumatriptan-induced coronary vasospasm, reports of sumatriptan-induced anomalous right coronary artery (RCA) vasospasm are rare. This case contributes to the literature by highlighting the unique presentation of an anomalous RCA vasospasm, as it may present with atypical symptoms such as variable chest pain patterns and transient ischemic episodes, which can lead to diagnostic confusion with other cardiac conditions, emphasizing the need for heightened clinical awareness and tailored diagnostic approaches. Here, we present the case of a 50-year-old female patient who exhibited ischemic changes on the EKG after receiving a sumatriptan injection. Notably, she had a normal EKG and routine cardiac catheterization before the medication.

## Case presentation

A 50-year-old woman with a medical history of hypertension and a family history of premature coronary artery disease (CAD), including CAD in her mother (when she was aged 59 years), complained of acute chest pain, an 8/10 pressure-like sensation (Wong-Baker FACES Pain Rating scale), and radiation to her jaw and upper extremities. Emergency Medical Services rushed the patient to the emergency department; en route, she took four baby aspirins before presentation to the hospital. The patient had no fever, chills, headache, blurred vision, shortness of breath, or syncope. She did not have any recent viral symptoms, and her social history was negative for smoking or alcohol intake. She mentioned that she had a similar chest pain episode earlier in the day, but it self-resolved within less than five minutes. Her subsequent symptoms developed abruptly and lasted for around 20 minutes without apparent causes. 

At presentation, her vital signs showed elevated blood pressure; her systemic examination was remarkable, only for mild tonsillar enlargement and erythema without exudate. Initial EKG revealed normal sinus rhythm with no ischemic changes and borderline or non-specific abnormalities. The laboratory results are summarized in Table 1. The complete blood count (CBC) and comprehensive metabolic panel were within the normal limits. Meanwhile, the initial troponin level was 4.9 picograms (pg)/mL (normal range 8.7-18.7 pg/mL; the 99th percentile cutoff for women is 11.8 pg/mL). At the outset, the patient's HEART (history, EKG, age, risk factors, and troponin) score was 4 points, indicating a moderate risk, and the risk of major adverse cardiovascular events ranged from 12% to 16.6%, for which the steps that follow include additional observation, serial high-sensitivity troponin (hs-cTn) measurements, and admission to observation units. 

**Table 1:** Laboratory findings

Tests	Day 1	Day 2	Day 3	Day 4
Hemoglobin (g/dl)	13.8	13.0	13.2	13.8
Platelets (x10^5^)	338	320	285	295
Sodium ((mmol/L)	136	137	135	136
Potassium (mmol/L)	3.8	3.9	4.3	3.8
Creatinine (mg/dL)	0.6	0.5	0.5	0.6
Troponin (pg/mL)	4.9	34.5	34.7	16.8
Low-density lipoprotein (mg/dL)	-	-	-	108.7

The initial screening for respiratory syncytial virus (RSV), COVID-19, influenza A, B, and *Streptococcus* rapid throat test was negative. Although the patient's initial chest pain subsided, the repeat troponin elevated to 34.5 pg/mL. She was transferred to telemetry status for further monitoring.

During her telemetry stay, Cardiology reviewed her case. Given the patient's significant family history of premature coronary artery disease and chest pain, the patient was started on anticoagulation using unfractionated heparin with the concerns of typical angina, and given the significant history of premature coronary artery disease, and was scheduled for coronary angiography. Echocardiography showed normal left ventricular systolic function, normal diastolic function with no regional wall abnormality, and valvulopathy. Similarly, we ordered the fasting lipid panel and glycated hemoglobin (HbA1C), revealing low-density lipoprotein cholesterol (LDL-C) levels of 108.7 mg/dl and 5.9%; we added statin for secondary prevention. Meanwhile, the troponin level stabilized at 34.7 pg/mL and downtrended to 16.8, suggesting demand ischemia.

**Figure 1:**
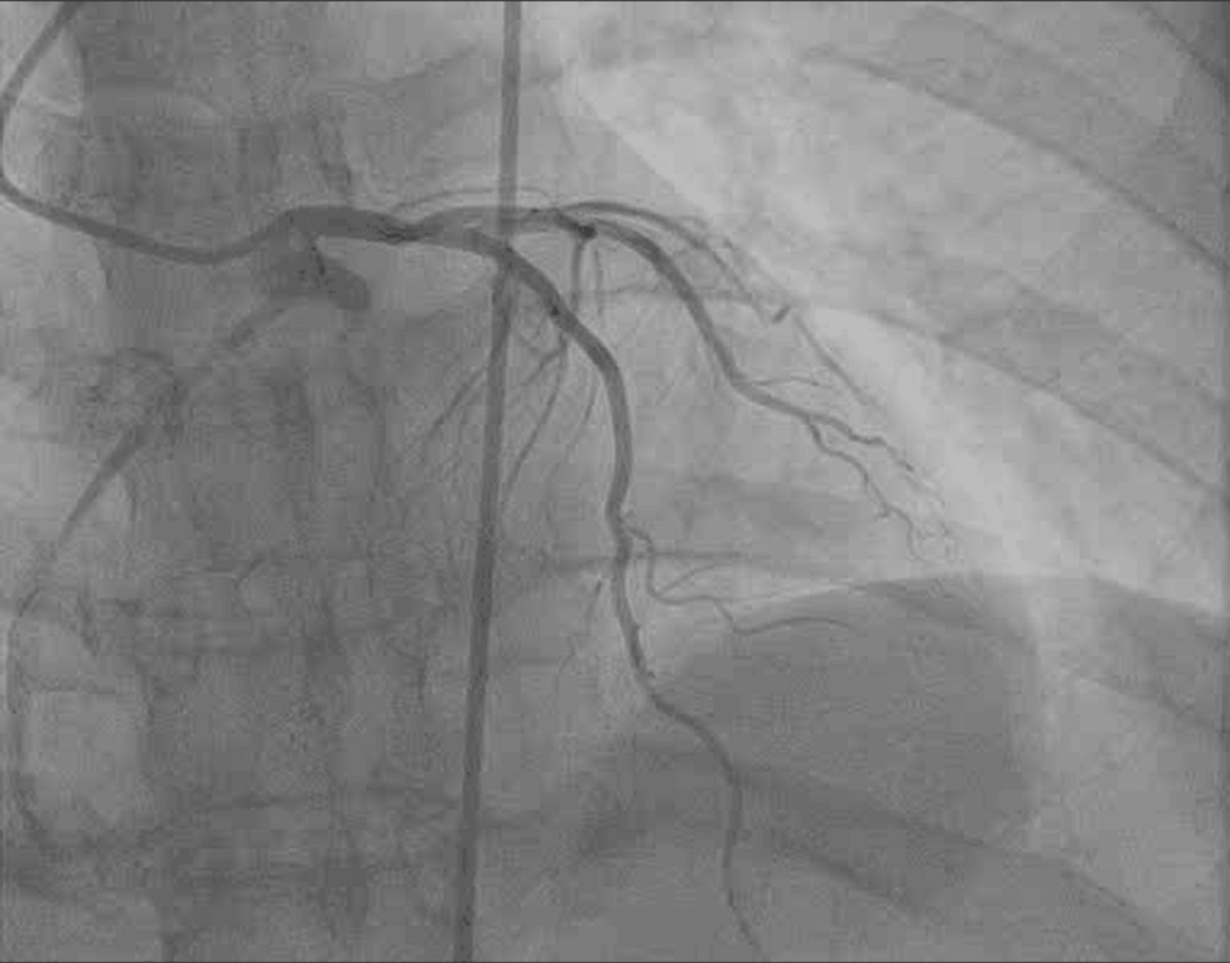
Coronary angiography reveals RCA with an anomalous origin emerging from the left coronary cusp RCA: right coronary artery

The patient underwent coronary angiography, which showed the RCA's anomalous origin from the left coronary cusp. There was a long, proximal RCA lesion (30% stenosis), which was generally considered incidental rather than functionally significant. The rest of the coronary system was angiographically normal, as shown in Figure 1. The patient returned to telemetry with no new findings.

**Figure 2:**
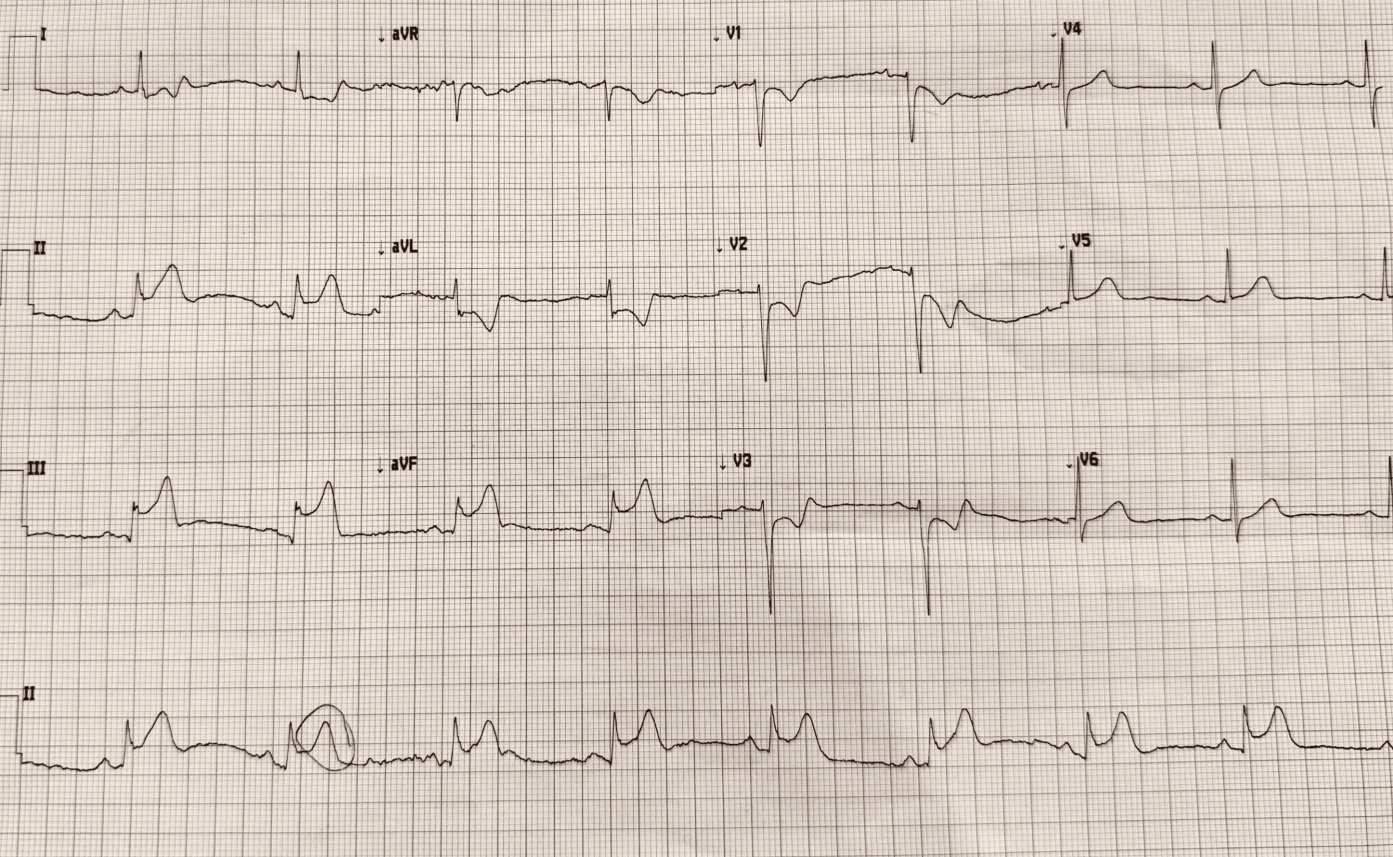
Electrocardiogram shows a new-onset ST elevation on the inferior leads, along with reciprocal changes in the precordial leads after taking the sumatriptan

**Figure 3:**
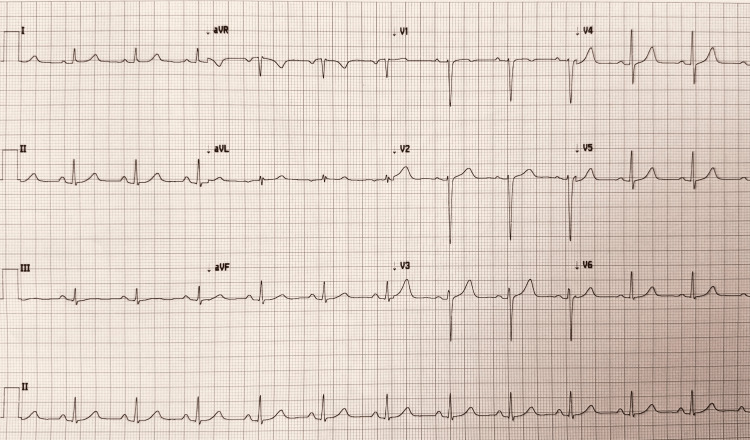
Electrocardiogram shows a normal sinus rhythm post nitrate treatment

We continued to treat the patient with dual antiplatelet agents, statins, beta-blockers, and angiotensin-converting enzyme (ACE) inhibitors. We also prescribed nitroglycerin as required for her chest pain. Given her recurrent unilateral headache, she was given a Fioricet. While this offered relief, her headache returned after two to three hours, and was provided a one-time dose of subcutaneous sumatriptan. Shortly after the sumatriptan, the patient reported 10/10 chest pain. The subsequent EKG revealed ST elevations on the inferior leads with reciprocal ST changes in the anterior leads, consistent with vasospasm rather than structural injury (Figure 2). One dose of sublingual nitroglycerin subsequently alleviated the pain, and the post-nitrate EKG displayed a normal appearance (Figure 3).

## Discussion

Triptans, selective 5-HT1B/1D agonists, are the cornerstone of migraine-specific prescription treatments in the United States. Few published case reports showed evidence of coronary vasospasm correlating with the use of either oral or intravenous sumatriptan [5,7,8]. Controlled trials have firmly established their efficacy for treating acute migraines [10,11]. In 2002, the American Headache Society's Triptan Cardiovascular Safety Expert Panel determined that triptans can be prescribed to patients at low risk for coronary artery disease without prior cardiac evaluation [11]. 

Data from clinical trials and post-marketing experience of sumatriptan in 2000 reported that through December 1998, around 451 serious cardiac events occurred within 24 hours of sumatriptan use in over 236 million migraine attacks [12]. In this review by Welch et al., they reported about 92 cardiac fatalities, highlighting the importance of careful patient selection. Sumatriptan has a half-life of about 90 minutes. Most patients experiencing serious cardiac events within one to three hours of taking sumatriptan had risk factors for coronary artery disease [12]. Therefore, the report suggests sumatriptan should be avoided in patients with history, symptoms, or signs of ischemic cardiac, cerebrovascular, or peripheral vascular syndromes. Although no recent or updated pharmacovigilance data is available, this older dataset provides insight into the correlation between triptans and cardiovascular side effects. 

Multiple published reports show a temporal relation between sumatriptan and MI. An overview of sumatriptan reported a sensation of chest pressure and tightness in 3-5% of patients, but there was no EKG evidence of ischemia [13]. Okonkwo et al. presented a case report of a patient without prior risk factors for CAD and angiographically unremarkable coronary arteries who presented with evidence of an AMI after oral sumatriptan use [14]. 

In contrast, in a large case-control study involving 63,575 migraine patients, 13,664 received a triptan prescription, and the analysis revealed no association between triptan use and MI (hazard ratio (HR) 0.93; 95%CI 0.60, 1.43) [15]. A randomized, double-blind, placebo-controlled trial assessed the effect of a 6-mg subcutaneous dose of sumatriptan on myocardial perfusion using 13NH3 PET. Nineteen healthy female migraineurs (ages 33-62) at low risk for ischemic heart disease showed no significant changes in myocardial perfusion; however, the study had limitations of low sample size, female gender only, and narrow age range [16]. Low risk is defined as having one or no risk factors, such as smoking, hypertension, high LDL cholesterol, low HDL cholesterol, a family history of early coronary heart disease, and age (men over 45, women over 55) [17]. Although these reports show no evidence of MI with sumatriptan, we should remain cautious.

Calcitonin gene-related peptide (CGRP) receptor antagonists such as rimegepant and ubrogepant, are effective in the acute treatment of migraine without causing vasoconstriction, which is a significant advantage for patients with cardiovascular contraindications to triptans [18].

Our case highlights the importance of thorough patient history and risk assessment before prescribing triptans, particularly in individuals with cardiovascular risk factors. 

## Conclusions

It is crucial to evaluate primary coronary risk factors, including diabetes, hypertension, smoking, obesity, and a strong family history of CAD. In patients with multiple cardiovascular risk factors who have a negative cardiovascular evaluation, it should be considered to administer the first dose in a medically supervised setting with an EKG immediately and periodic evaluation in long-term users. To ensure patients are well-informed about potential cardiac risks when using sumatriptan, standardized educational materials, which include cardiovascular risk information, patient labeling, promptly reported symptoms, including chest pain or tightness, and periodic evaluation, should be considered. We should tailor the frequency and type of follow-up assessments considering the individual patient's risk factors and clinical presentation, which includes follow-up EKG, lipid profile, blood pressure, and additional testing, including stress testing and coronary calcium scoring in certain high-risk patients with multiple coronary risk factors.

Furthermore, we strongly discourage the use of sumatriptan in patients who have a documented history of symptomatic CAD along with anomalous RCA and vasospasm, as seen in this case. In addition to that, all the sumatriptan-advised patients need to be informed about the possibility of MI so that they can be cautious and seek medical attention if they experience any chest pain or tightness.
